# An ureido-substituted benzenesulfonamide carbonic anhydrase inhibitor exerts a potent antitumor effect in vitro and in vivo

**DOI:** 10.1186/s40164-025-00690-z

**Published:** 2025-08-08

**Authors:** Giorgia Gazzaroli, Camilla Tavani, Serena Filiberti, Maria Luisa Massardi, Marta Turati, Giulia Garattini, Thomas S. Peat, Giuseppe Pieraccini, Andrea Angeli, Claudiu T. Supuran, Fabrizio Carta, Arianna Giacomini, Roberto Ronca

**Affiliations:** 1 Department of Molecular and Translational Medicine, University of Brescia, Brescia, 25123 Italy; 2 School of Biotechnology and Biomolecular Sciences, UNSW, Sydney, 2052 Australia; 3 CISM - Mass Spectrometry Centre University of Florence, via U. Schiff 6, Sesto Fiorentino, 50019 Italy; 4 Department of NEUROFARBA - Section of Pharmaceutical and Nutraceutical Sciences, University of Florence, via U. Schiff 6, Sesto Fiorentino, 50019 Italy

**Keywords:** Carbonic anhydrase inhibitor, Cancer therapy, Metastasis, Benzenesulfonamide

## Abstract

Carbonic anhydrase IX (CA IX) is a tumor-specific metallo-enzyme upregulated during hypoxic conditions and implicated in several pathophysiological processes where tissue pH regulation is required such as cancer, cell invasion, metastatic and stem-like features, drug resistance and recurrence. Indeed, CA IX expression has been correlated with poor prognosis, aggressiveness and disease progression in several solid tumors, and its targeting has been proposed as a therapeutic approach to treat aggressive cancers. To date, several CA IX targeting approaches have been developed to inhibit its activity in neoplastic tissues including the clinical grade (Phase Ib/II) ureido-substituted benzenesulfonamide SLC-0111, which has been widely investigated over the past years. In this study, we carried out a detailed characterization of a SLC-0111 derivative, FC-531, evaluating its anti-tumor potential in parallel with SLC-0111 on a panel of human cell lines representative of different cancer types. Finally, we evaluated the safety profile of FC-531 in vivo and demonstrated its capacity to reduce tumor growth and metastatization in vivo. Together, our data provide the rationale for the exploitation of FC-531 as a potent CA IX inhibitor for the management of different CA IX- expressing solid tumors.

**Supplementary Information:**

The online version contains supplementary material available at 10.1186/s40164-025-00690-z.

To the Editor,

Human (h) Carbonic Anhydrases (CA) are zinc metalloenzymes that reversibly catalyse the hydration of CO_2_ to bicarbonate and protons. Among all CAs, the IX has emerged as a tumor-associated cell-surface CA isoform mainly induced by hypoxia and involved in the adaptation of tumor cells to acidosis [1]. CA IX has been found overexpressed in several solid tumors, but not in healthy tissues except for epithelial cells of the stomach and the gut. In tumor cells, its enzymatic activity contributes to the balancing of the cellular pH gradient allowing the generation of an acidic extracellular pH (pH_e_) and a slightly more alkaline intracellular/cytosolic pH (pH_i_) [2], and this has been associated to increased cancer cells invasion and metastasis as well as to induction of stem-like features, drug resistance and recurrence [3, 4]. Accordingly, the expression of CA IX has been correlated with poor prognosis, aggressiveness and disease progression in several solid tumors [5, 6], and its targeting has been proposed as a therapeutic approach to treat aggressive cancers [4, 7, 8].

CA IX targeting agents able to block its activity in neoplastic tissues have been developed over the last decade, including a sulfonamide CA IX inhibitor (SLC-0111) and an antibody (CA9hu-1) that entered a Phase Ib/II clinical trials for the treatment and imaging of different solid tumors [9, 10].

Given the relevance of CA IX in cancer, several synthetic approaches have been applied to realize new inhibitors from different chemical backbone and also exploiting SLC-0111 as a lead compound [11]. Among the ureido-substituted benzenesulfonamides class that included SLC-0111, a structurally related compound, here named FC-531 (4-CF_3_C_6_H_4_) was originally synthesized and displayed high activity for inhibiting CA IX [8].

Here, we carried out a deeper characterization of FC-531, studying its interaction with CA IX and evaluating its anti-tumor potential in parallel with SLC-0111.

In vitro, FC-531 displayed high inhibition potencies for the transmembrane and tumor-associated isoforms CA IX and CA XII (i.e. *K*_Is_ = 6.2 nM and 2.3 nM, respectively; Table S1), in line with those obtained for the reference compound SLC-0111 (i.e. *K*_I_s = 45.1 nM and 4.5 nM, respectively) [8]. It is worth noting they differentiate for the activity on the physiologically relevant CA I and II isoforms, abundantly expressed at cytosolic level in both healthy and tumor cells. Indeed, FC-531 showed remarkable efficacy in inhibiting the hCA I isoform (*K*_I_ = 9.7 nM) while was far less effective on the hCA II (i.e. *K*_I_ = 1150 nM). The peculiar kinetic trend of FC-531 is of high interest as it paves the way for the development of small molecules potentially useful for the management of hypoxic cancers by recruiting CA isoforms (i.e. I and II) physiologically cooperative with those primarily associated to the disease [12]. The binding mode of FC-531 was assessed in complex with hCAs II and IX by means of X-ray crystallography (Fig. 1A-C). As shown in Fig. 1B, superposition of the 2 structures clearly showed an almost complete matching of the ligand bindings, the main differences in binding interactions being spotted at the upper part of the enzymatic cleft being the Phe131 residue mainly responsible for pushing the inhibitor away with beneficial effects for the key interaction among the ligand ureidic oxygen with the Val131 residue [13].

**Fig. 1:**
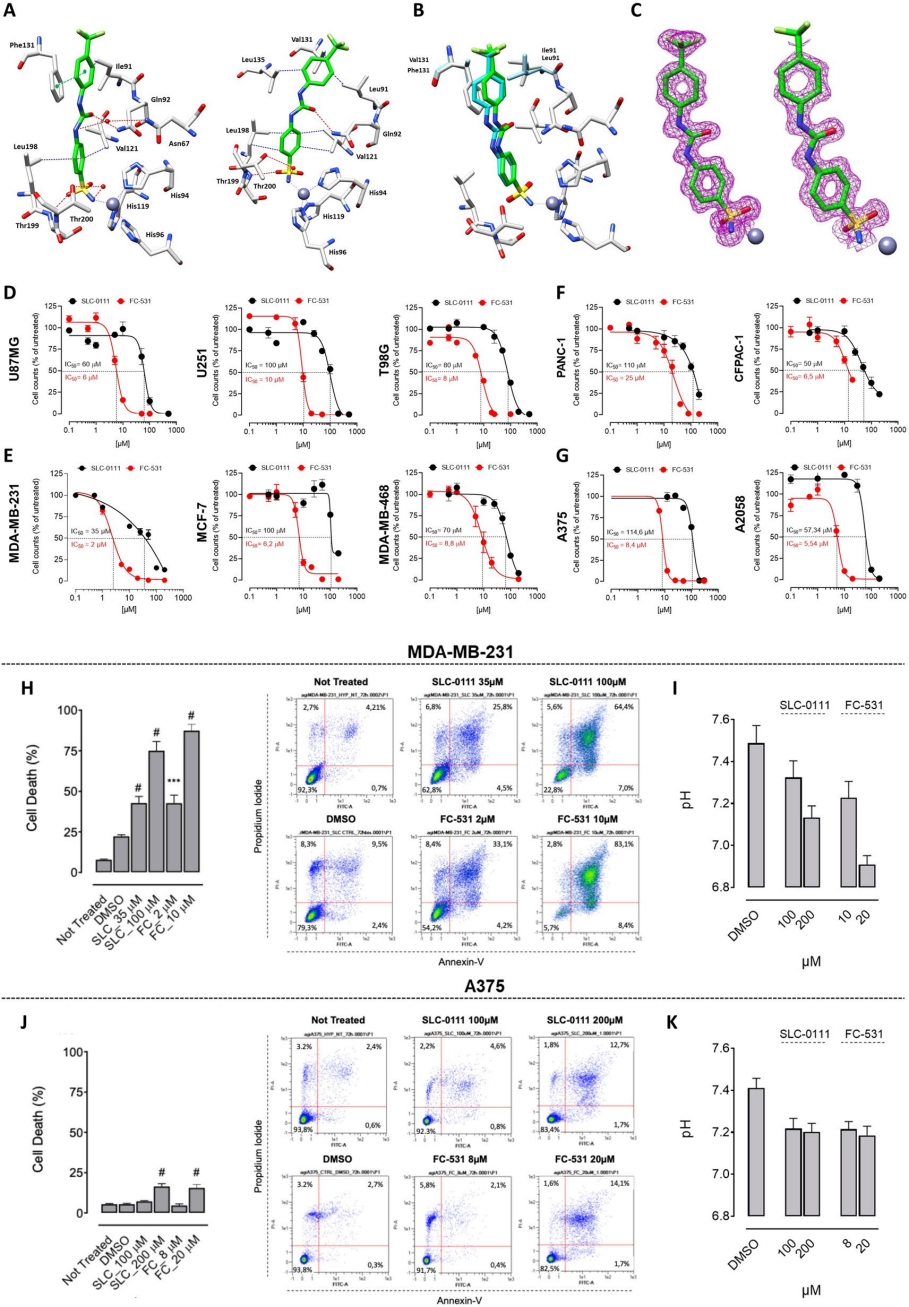
**A**) Zoomed-in view of FC-531 in complex with (left) hCA II (PDB 8UFX), (right) hCA IX-mimic (PDB 8UFW). **B**) Overlay of FC-531 bound to hCA II and IX-mimic; **C**) Electron density maps of FC-531 in complex with hCAs II (left) and IX-mimic (right) respectively. Residues involved in the inhibitor–enzyme binding are shown as sticks and labelled. Hydrogen bonds and water bridges are depicted as red dotted lines, whereas van-der Waals as blue dotted lines. The grey sphere represents the Zinc (II) ion in the protein active site. **D-G**) Cell viability of a panel of human cell lines representative of different cancer types after treatment with CA IX inhibitors, SLC-0111 (black) and FC-531 (red), for 72 h in hypoxia. The mean ± SEM is reported and the relative IC_50_ indicated for each treatment. Flow cytometry analysis of apoptotic cell death in (**H**) MDA-MB-231 and (**J**) A375 cells treated with SLC-0111 and FC-531 for 72 h in hypoxic conditions. Quantification of intracellular pH variation assessed by BCFL-AM pH sensitive probe in (**I**) MDA-MB-231 and (**K**) A375 cells after treatment with CA IX inhibitors SLC-0111 and FC-531 in hypoxic conditions. Data are the Mean ± SEM ***, *P* < 0.001; #, *P* < 0.0001

CA IX and CA XII have been reported to be upregulated in tumor contexts both in response to hypoxia and by the activation of oncogenic/mitogenic pathways [4]. Accordingly, we observed a significant increase of *CA9* and *CA12* genes expression in a panel of human cancers representing human glioblastoma (U87MG, U251 and T98G cells), breast cancer (MDA-MB-231, MCF-7, and MDA-MB-468 cells), pancreatic cancer (CFPAC-1 and PANC-1 cells), and melanoma (A375 and A2058 cells) cultured in hypoxic conditions (Figure S1). On this base, the anti-tumor effect of FC-531, in parallel with SLC-0111 as a reference, was assessed on the same human cancer cell lines under hypoxic conditions that better mimic the tumor milieu and increase CA IX expression. As shown in Fig. 1D-G, both CA inhibitors significantly reduced the cell growth capacity of all cancer cell lines tested (Fig. 1D-G). This effect was maintained, even if with higher IC_50_ values, also in normoxic conditions (Figure S2), in line with the basal expression of CA IX and XII in tumor cells. In keeping with these data, annexinV/propidium iodide (PI) staining revealed that treatment with FC-531 and SLC-0111 significantly triggers apoptotic cell death on prototypic triple negative breast cancer (TNBC) and melanoma cells (Fig. 1H, J), and this was paralleled by the capacity to reduce/acidify the pH_i_ in both cell lines as a result of CA inhibition (Fig. 1I, K). Notably, no significant toxic effects on non-tumor cells (i.e. human skin fibroblasts and peripheral blood mononuclear cells/PBMCs; Figure S3) were observed. Altogether, in vitro FC-531 displayed a higher anti-tumor effect in comparison with SLC-0111, thus suggesting a promising anti-tumor profile.

Before proceeding with anti-tumor evaluation in vivo, we characterized the bioavailability of FC-531 in the plasma after both intra-peritoneal (i.p.) and oral administration in mice (Figure S4A), and confirmed that treatments with the selected dose of 50 mg/kg (already demonstrated to be active for SLC-0111 in the treatment of various tumor types [14, 15]) was well tolerated as shown by no variations in terms of body weight (Figure S4B) and haematological and biochemical blood parameters (Figure S4C).

The therapeutic potential of FC-531 was then evaluated in vivo in orthotopic xenograft models of human TNBC (MDA-MB-231) and melanoma (A375) in mice (Fig. 2) and in a syngeneic model of murine melanoma (B16F10) (Figure S5). As shown in Fig. 2A-B, FC-531 exerted a significant anti-tumor effect in both xenograft models, and the efficacy was comparable with the effect exerted by the clinical grade SLC-0111 drug. Immunohistochemical analysis of the explanted tumors confirmed the expression of CAIX by tumor cells and that treatment with CA inhibitors significantly reduced tumor cell proliferation (pHH3 staining), triggered tumor cell apoptosis (cleaved-caspase 3 staining) and reduced tumor angiogenesis (CD31 staining) (Fig. 2C). Finally, the therapeutic potential was confirmed in a TNBC-derived lung metastases model where both FC-531 and SLC-0111 significantly reduced the metastasis burden in the lung in comparison with control group (Fig. 2D).

Given the relevance of CA IX in cancer, the continuous development and refinement of CA IX inhibitors is regarded as a promising approach in cancer therapy and new drugs have been developed. Here, we focused on the sulfonamide CA IX inhibitor SLC-0111, and its class analog FC-531 that was initially synthesized together with SLC-0111. Altogether, our data provide a finer characterization of the CA IX inhibitor FC-531 as a potential SLC-0111 peer endowed with promising anti-neoplastic activity both in vitro and in vivo in different tumor types. On this basis future investigation will further characterize FC-531 mechanistic activity and will improve formulation and treatment schedule to pave the way for its potential exploitation in future clinical settings and to continue the refinement of small molecules CA IX inhibitors.

**Fig. 2:**
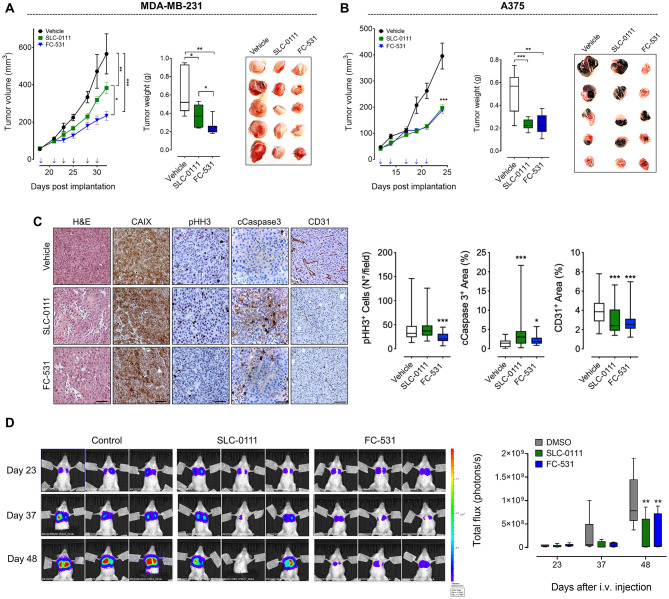
**A)** Tumor growth and weight of MDA-MB-231 cells orthotopically injected into the mammary fat pad and treated (blue arrows) with SLC-0111, FC-531 or vehicle. **B)** Tumor growth and weight of A375 cells injected subcutaneously and treated (blue arrows) with SLC-0111, FC-531 or vehicle. **C)** Immunohistochemical analysis of MDA-MB-231 tumors harvested at the end of the experiment. Quantification of pHH3-, cCaspase3- and CD31- positive cells was carried out by ImageJ software. Scale bar 100 μm (H&E, CAIX, pHH3 and cCaspase3) and 200 μm (CD31). In box and whiskers graphs, boxes extend from the 25th to the 75th percentiles, lines indicate the median values, and whiskers indicate the range values. **D**) Luciferase expressing MDA-MB-231 cells were injected intravenously in NOD/SCID mice to obtain lung metastasis and mice were treated every other day with SLC-0111 (50 mg/kg), FC-531 (50 mg/kg), or control/vehicle DMSO. Representative images of bioluminescence imaging (left) and quantification (right) of lung metastasis are reported. Data are the Mean ± SEM; *, *P* < 0.05; **, *P* < 0.01 ***, *P* < 0.001

## Electronic supplementary material

Below is the link to the electronic supplementary material.

Supplementary Material 1

## Data Availability

No datasets were generated or analysed during the current study.
