# Supplementary material for: An ureido-substituted benzenesulfonamide carbonic anhydrase inhibitor exerts a potent antitumor effect in vitro and in vivo
Source: Exp Hematol Oncol. 2025 Aug 8;14:104. doi: 10.1186/s40164-025-00690-z (PMC12333284; doi:10.1186/s40164-025-00690-z)
Supplement: Supplementary file 1 — Supplementary Material 1 [file 40164_2025_690_MOESM1_ESM.pdf]

SUPPLEMENTARY INFORMATION

Table S1. Inhibition of cytosolic hCAs I, II and membrane-associated IX, XII by the CO_2 hydrase Stopped-Flow assay of SLC-0111, FC-531 and Acetazolamide (AAZ) as a reference drug [1].

Compound	K_I (nM)* [1]			
	**hCA I**	**hCA II**	**hCA IX**	**hCA XII**
**SLC-0111**	5080	960	45.1	4.5
**FC-531**	9.7	1150	6.2	2.3
**AAZ**	250.0	12.1	25.6	5.7

* Mean from 3 different assays, by the stopped-flow technique (errors were in the range of ± 5-10 % of the reported values).

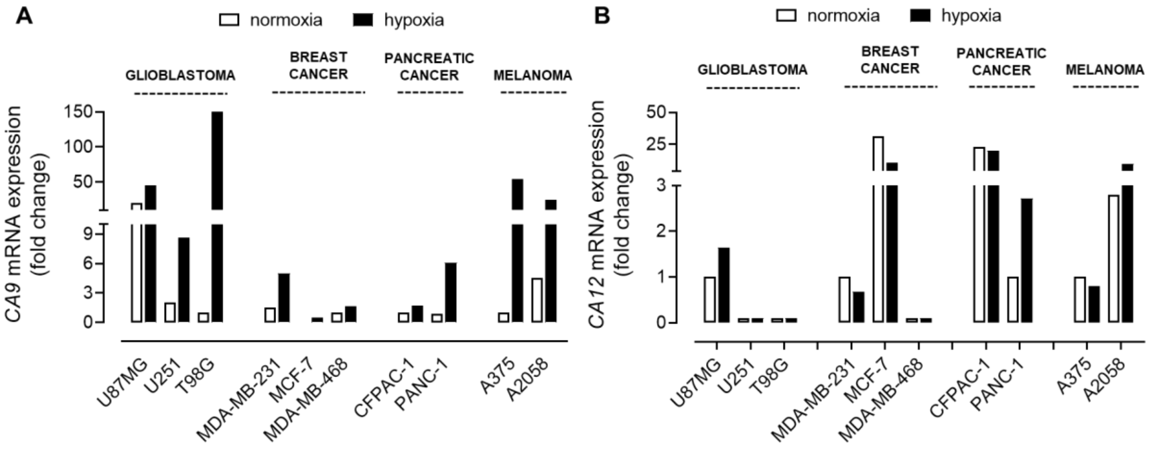

Figure S1: qPCR analysis for the expression level of *CA9* (**A**) and *CA12* (**B**) genes in a panel of human cell lines representative of different cancer types cultured in normoxic or hypoxic conditions for 72 hours.

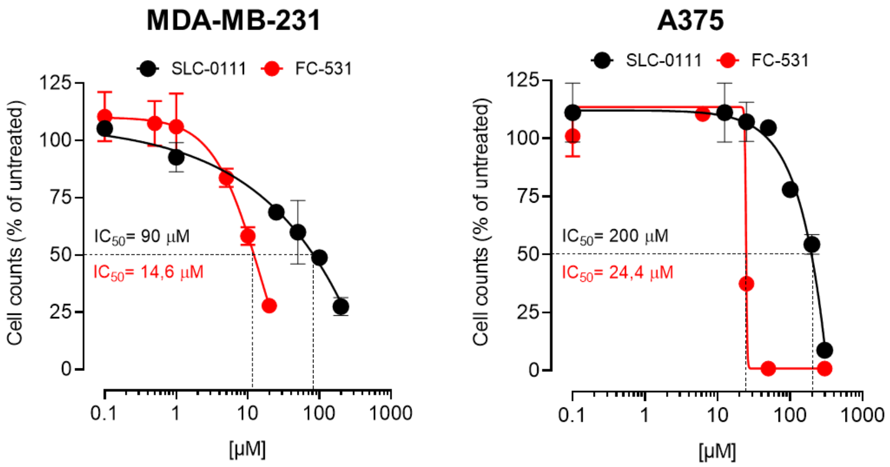

Figure S2: Cell viability of MDA-MB-231 and A385 cells after treatment with CA IX inhibitors, SLC-0111 (black) and FC-531 (red), for 72 hours in normoxia. The mean ± SEM is reported and the relative IC_{50} indicated for each treatment.

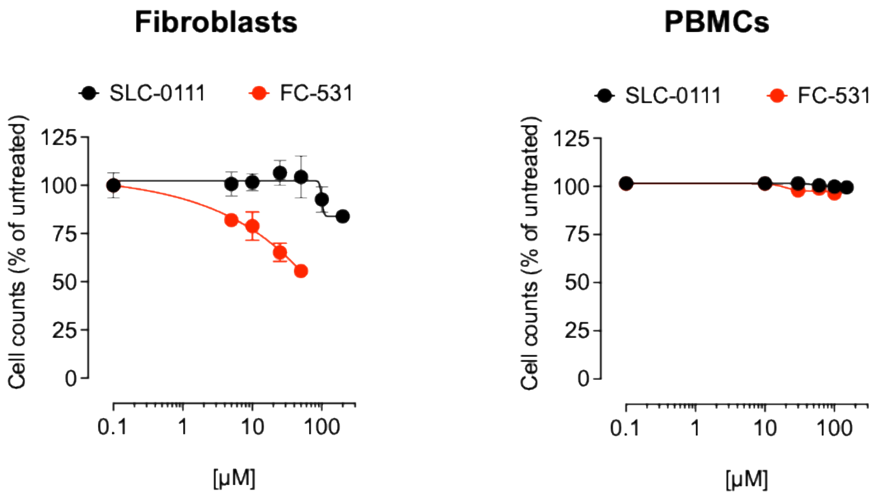

Figure S3: Cell viability of human fibroblasts and PBMCs after treatment for 72 hours with CA IX inhibitors, SLC-0111 (black) and FC-531 (red). Data are mean ± SEM.

A

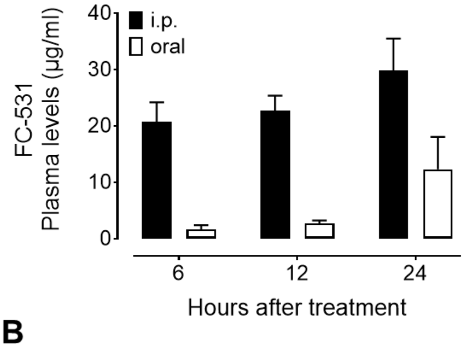

B

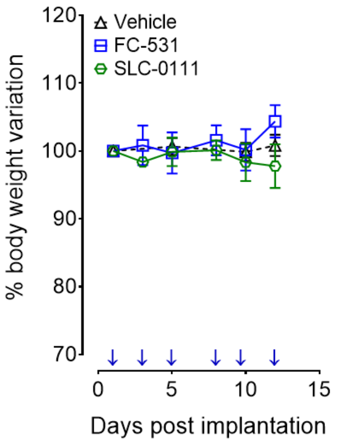

C

BLOOD PARAMETERS	Control mice	FC-531 treated
White blood cells (103/µL)	5.61 ± 1.00	5.79 ± 1.62
Neutrophils (K/uL)	0.82 ± 0.18	0.83 ± 0.24
Neutrophils (%)	14.83 ± 3.61	14.40 ± 0.14
Lymphocytes (K/µL)	4.30 ± 1.04	4.55 ± 1.28
Lymphocytes (%)	76.03 ± 7.82	78.50 ± 0.14
Monocytes (K/µL)	0.40 ± 0.15	0.29 ± 0.10
Monocytes (%)	7.50 ± 4.00	4.95 ± 0.35
Eosinophils (K/µL)	0.09 ± 0.07	0,12 ± 0.01
Eosinophils (%)	1.50 ± 0.96	2.05 ± 0.49
Basophils (K/µL)	0.01 ± 0.01	0.01 ± 0.01
Basophils (%)	0.13 ± 0.12	0.10 ± 0.14
Red blood cells (106/µL)	7.41 ± 0.41	8.78 ± 0.45
Hemoglobin (g/dL)	10.83 ± 0.61	12.40 ± 0.71
Hematocrit (%)	31.13 ± 2.25	35.35 ± 1.91
Mean Corpuscular Volume (fL)	41.97 ± 1.76	40.30 ± 0.14
Mean corp Hemoglobin (pg)	14.60 ± 0.10	14.15 ± 0.07
Mean Corp Hemoglobin (g/dL)	34.83 ± 1.26	35.05 ± 0.07
RDW (%)	14.80 ± 1.04	15.45 ± 0.78
Platelets (10^3/µL)	928.67 ± 315.84	1077.00 ± 79.20
**BIOCHEMICAL PARAMETERS**	**Control mice**	**FC-531 treated**
ALT (U/L)	37.33 ± 8.33	34.00 ± 2.83
AST (U/L)	121.33 ± 16.17	98.00 ± 8.49
Albumin/globulin (A/G) ratio	1.37 ± 0.26	1.70 ± 0.04
Urea (mmol/L)	6.53 ± 0.23	9.80 ± 0.28
Albumin (g/L)	32.80 ± 0.69	39.80 ± 0.85
Total proteins (mg/dL)	57.20 ± 3.67	63.20 ± 1.13
Phosphorus (mmol/L)	2.53 ± 0.23	3.20 ± 0.00
Calcium (mmol/L)	2.37 ± 0.12	2.22 ± 0.03
Potassium (mmol/L)	6.93 ± 0.12	9.22 ± 0.31
Creatine kinase (U/L)	796.00 ± 588.95	454.00 ± 178.19
Creatinine (µmol/L)	19.07 ± 3.33	35.20 ± 6.79
LDH (U/L)	1150.67 ± 202.56	2156.00 ± 135.76
Triglycerides (mmol/L)	1.20 ± 0.11	1.64 ± 0.11
Iron (µmol/L)	23.20 ± 1.44	27.20 ± 2.26

Figure S4. A) Plasma levels of FC-531 after intraperitoneal (i.p) and oral administration in mice. **B**) Body weight of animals monitored during treatment with SLC-0111 and FC-531. Blue arrows indicate the day of treatment. **C**) At the end of the treatments (2 weeks), whole blood and serum from FC-531 treated animals was collected and analysed in terms of blood cell composition and biochemical parameters, respectively. Data are mean ± SEM (n = 4-5 mice/group).

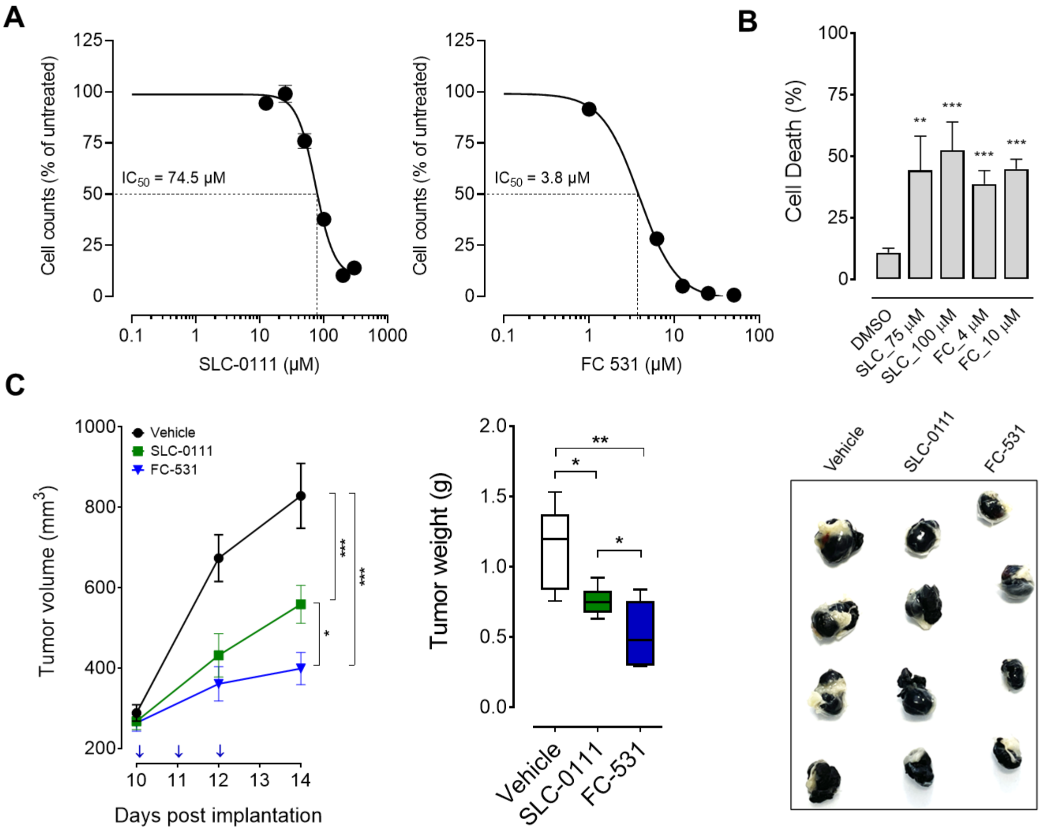

Figure S5. A) Cell viability of murine melanoma B16F10 cells treated with CA IX inhibitors, SLC-0111 and FC-531, for 72 hours in hypoxia. **B**) Flow cytometry analysis of apoptotic cell death in SLC-0111 and FC-531-treated B16F10 cells for 72 hours. **C**) Tumor growth and weight of B16F10 cells injected subcutaneously and treated (blue arrows) with SLC-0111, FC-531 or vehicle. *, $P < 0.05$; **, $P < 0.01$ ***, $P < 0.001$.

SUPPLEMENTARY MATERIALS AND METHODS

Crystallization and structure determination

CA II was purified as previously described [2]. The protein was set up in MRC-2 plates (Molecular Dimensions, Sheffield, UK) using a Phoenix robot (Art Robbins Instruments, Sunnyvale, CA, USA) in 250 nL plus 250 nL drops with the protein at 4.2 mg/mL with the reservoir solutions being 2.5 to 2.8 M ammonium sulfate and 50 to 100 mM tris buffer pH 8.1 to 8.9. Crystals grew over the course of 1-3 days at 8 C. Crystals were soaked with compounds for 24 hours before harvesting with glycerol (20% final concentration) as a cryoprotectant and frozen in liquid nitrogen.

The CA IX mimic protein was purified as previously described [3]. The protein was set up in MRC-2 plates in 100 nL plus 100 nL drops with the protein at 5 mg/mL with reservoir solutions being 2.6 to 2.9 M ammonium sulfate and 100 mM tris buffer at pH 8.3 to 8.9. Crystals grew over the course of several days at 20 C. The crystals were soaked with compounds for 24 hours before harvesting with glycerol (20% final concentration) as a cryoprotectant and frozen in liquid nitrogen.

Data were collected at the Australian Synchrotron MX2 beamline [4]. The data were processed with XDS [5], scaled with Aimless [6] and the structures were phased using Phaser [7] with 7MU3 used as a starting model for the CA9 mimic and 4CQ0 for CAII. The data were manually rebuilt using Coot [8] and refined using REFMAC [9]. Dictionary files for the compounds were generated in eLBOW [10] and initially placed using LigandFit [11] as part of the Phenix software package. See also Supplementary Table 1 for X-ray statistics.

Reagents and cell cultures.

Human U87MG, U251, T98G glioblastoma cells, human MDA-MB-231, MCF-7, MDA-MB-468 breast cancer cells and human A2058 melanoma cells were purchased from American Type Culture Collection (ATCC) and cultured in DMEM plus 10% FBS; human PANC-1, CFPAC-1 ductal pancreatic adenocarcinoma cells and human A375 melanoma cells were obtained from American Type Culture Collection (ATCC) and cultured in RPMI plus 10% FBS. All cells were maintained at low passage, returning to original frozen stocks every 3 to 4 months. Finally, cells were incubated at 37°C in normoxic condition (21% O2, 5% CO_2 and N_2 for the rest) or hypoxic condition (1% O_2, 5% CO_2 and N_2 for the rest) and tested regularly for Mycoplasma negativity by PCR and DAPI staining. Compounds SLC-0111 and FC- 531 were synthesized as previously described in [1] by dr. C. T. Supuran (University of Florence).

RT-qPCR.

Total RNA was extracted using TRIzol Reagent (Invitrogen) according to manufacturer's instructions. Two micrograms of total RNA were retrotranscribed with MMLV reverse transcriptase (Invitrogen)

using random hexaprimers. Then, cDNA was analyzed by quantitative PCR using primers for the following targets (Invitrogen): human CA IX *For: CTTTGAATGGGCGAGTGATT, Rev: TTCTGTGCTGCCTTCTCATCT*; human CA XII *For: TGGGCATCATCCTCTCACT, Rev: GCCTCAGTCTCCATCTTGGT*. Human GAPDH (*For: TGCCATCACTGCCACCCAGA, Rev: CGCGGCCATCACGCCACAG*) was used as housekeeping gene.

In vitro assays.

Cell proliferation. Cells were seeded in 96-well culture plates, treated with compounds and cultured under hypoxia conditions for 72 hours. Propidium iodide staining (Immunostep, Salamanca, SP, EU) was used to detect PI viable cells by flow cytometry. Absolute cell counts were obtained by the counting function of the MACSQuant® Analyzer (Miltenyi Biotec).

Apoptosis assay. Cells were treated with compounds and cultured under hypoxia conditions for 72 hours. Apoptotic cell death was assessed by Annexin-V/Propidium Iodide double staining (Immunostep) according to manufacturer's instructions.

pH variation assay. Cells were seeded under hypoxia conditions overnight and treated with compounds for 10 minutes. Intracellular pH was then assessed through Fluorometric Intracellular pH Assay Kit (Sigma-Aldrich) according to manufacturer’s instructions.

In vivo studies.

In vivo experiments were performed in compliance with the Italian laws (D.L. 116/92 and following additions) that enforce the EU 86/109 Directive and were approved by the local animal ethics committee (OPBA, Organismo Preposto al Benessere degli Animali, Università degli Studi di Brescia, Italy).

Bioavailability and toxicity study. For the determination of plasma levels of FC-531, eight-week-old C57BL/6 mice were treated i.p. or orally with 50 mg/kg of the compound and blood samples were collected after 6, 12, and 24 hours. Plasma was prepared and analysed by mass spectrometry for quantification of FC-531. For haematological and biochemical blood analyses, C57BL/6 mice were treated every other day i.p. with 50 mg/kg of FC-531. After two weeks of treatment, blood was collected and analyses performed at the Ististuto Zooprofilattico Sperimentale della Lombardia e dell’Emilia Romagna (IZSLER).

Heterotopic and orthotopic tumor models. Six- to 8-week-old female NOD/SCID mice (Envigo) were orthotopically injected into the mammary fat pad with $3x10^6$ cells MDA-MB-231 cells in 50 μL of PBS. For A375 cells, NOD/SCID females were injected subcutaneously with $3x10^6$ cells/mouse in 100 μL of PBS, and for B16F10 cells, C57BL/6 females were injected subcutaneously with $5x10^5$ cells/mouse in 100 μL of PBS. When tumors were palpable, mice were randomly assigned to receive i.p. treatment every other day with SLC-0111 (50 mg/kg), FC-531 (50 mg/kg), or control/vehicle

DMSO. Tumor volume was measured with caliper (range: 0-150 mm; resolution: 0.01 mm) and calculated according to the formula $V=(Dxd^2)/2$, where D and d are the major and minor perpendicular tumor diameters, respectively. At the end of the experimental procedure, tumor nodules were harvested, their volume and weight were analysed and finally they were processed for histologic analysis.

Metastasis model. For the metastasis model, six- to 8-week-old NOD/SCID female mice (Envigo) were injected intravenously with $5x10^5$ cells MDA-MB-231*luc* cells in 50 µL of PBS. Fourteen days after tumor cells injection, mice were imaged for the expression of luciferase in terms of bioluminescence (BLI) using an IVIS Lumina III apparatus (PeckinElmer) after receiving an i.p. injection with 150 µL of D-Luciferin (PeckinElmer). Mice were then divided into experimental groups to receive i.p. treatment every other day with SLC-0111 (50 mg/kg), FC-531 (50 mg/kg), or control/vehicle DMSO. Longitudinal imaging was performed to follow the metastasis/BLI growth in the lungs. Analysis of the BLI was performed using the Living Image™ Software.

Immunohistochemical analyses.

For IHC on tumor xenograft samples, formalin-fixed and paraffin-embedded samples were sectioned at a thickness of 3 µm. After that, they were dewaxed, hydrated, and processed for immunohistochemistry, mouse anti-human Carbonic Anhydrase IX [M75] provided by S. Pastorekova, rabbit anti-human phospho-Histone H3 (Merck Millipore), rabbit Anti-cleaved Caspase-3 (Cell Signaling) antibodies or rat anti-CD31 (Dianova) rabbit. Positive signal was revealed by 3,3'-diaminibenzidine (Roche) stainings. Finally, sections were counterstained with Mayer hematoxylin before analysis by light microscopy. Images were obtained through the high-resolution and automatic scanner Aperio System (Leica Biosystems, Wetzlar, Germany, EU) and image analysis was carried out using the opensource ImageJ software.

Statistical analyses.

Statistical analyses were carried out using GraphPad Software Prism 8. Student t-test for two-tailed unpaired data was used to test the probability of significant differences between two groups of samples. For more than two groups of samples, data were analysed with a one-way ANOVA and corrected by the Bonferroni multiple comparison test. Tumor volume data were analysed with a two-way ANOVA and corrected by the Bonferroni test. Unless otherwise specified, differences were considered significant when $p < 0.05$.

References.

[1] F. Pacchiano, F. Carta, P.C. McDonald, Y. Lou, D. Vullo, A. Scozzafava, S. Dedhar, C.T. Supuran, Ureido-substituted benzenesulfonamides potently inhibit carbonic anhydrase IX and show antimetastatic activity in a model of breast cancer metastasis. Journal of medicinal chemistry 54 (2011) 1896-1902.

[2] J. Moeker, B.P. Mahon, L.F. Bornaghi, D. Vullo, C.T. Supuran, R. McKenna, S.A. Poulsen, Structural insights into carbonic anhydrase IX isoform specificity of carbohydrate-based sulfamates. Journal of medicinal chemistry 57 (2014) 8635-8645.

[3] P. Mujumdar, K. Teruya, K.F. Tonissen, D. Vullo, C.T. Supuran, T.S. Peat, S.A. Poulsen, An Unusual Natural Product Primary Sulfonamide: Synthesis, Carbonic Anhydrase Inhibition, and Protein X-ray Structures of Psammaplin C. Journal of medicinal chemistry 59 (2016) 5462-5470.

[4] N.P. Cowieson, D. Aragao, M. Clift, D.J. Ericsson, C. Gee, S.J. Harrop, N. Mudie, S. Panjikar, J.R. Price, A. Riboldi-Tunnicliffe, R. Williamson, T. Caradoc-Davies, MX1: a bending-magnet crystallography beamline serving both chemical and macromolecular crystallography communities at the Australian Synchrotron. Journal of synchrotron radiation 22 (2015) 187-190.

[5] W. Kabsch, Xds. Acta crystallographica. Section D, Biological crystallography 66 (2010) 125-132.

[6] P.R. Evans, G.N. Murshudov, How good are my data and what is the resolution? Acta crystallographica. Section D, Biological crystallography 69 (2013) 1204-1214.

[7] A.J. McCoy, R.W. Grosse-Kunstleve, P.D. Adams, M.D. Winn, L.C. Storoni, R.J. Read, Phaser crystallographic software. Journal of applied crystallography 40 (2007) 658-674.

[8] P. Emsley, K. Cowtan, Coot: model-building tools for molecular graphics. Acta crystallographica. Section D, Biological crystallography 60 (2004) 2126-2132.

[9] A.A. Vagin, R.A. Steiner, A.A. Lebedev, L. Potterton, S. McNicholas, F. Long, G.N. Murshudov, REFMAC5 dictionary: organization of prior chemical knowledge and guidelines for its use. Acta crystallographica. Section D, Biological crystallography 60 (2004) 2184-2195.

[10] N.W. Moriarty, R.W. Grosse-Kunstleve, P.D. Adams, electronic Ligand Builder and Optimization Workbench (eLBOW): a tool for ligand coordinate and restraint generation. Acta crystallographica. Section D, Biological crystallography 65 (2009) 1074-1080.

[11] C.M. Venkatachalam, X. Jiang, T. Oldfield, M. Waldman, LigandFit: a novel method for the shape-directed rapid docking of ligands to protein active sites. Journal of molecular graphics & modelling 21 (2003) 289-307.
